# Acne Keloidalis Nuchae: A Staged Reconstruction

**DOI:** 10.7759/cureus.18173

**Published:** 2021-09-21

**Authors:** Amir Labib, Louay Salfity, Barry Powell

**Affiliations:** 1 Plastic Surgery, St. George’s University Hospitals NHS Foundation Trust, London, GBR; 2 Plastic and Reconstructive Surgery, St. George’s University Hospitals NHS Foundation Trust, London, GBR

**Keywords:** treatment, therapy, npwt, hair disorder, scalp, acne keloidalis nuchae

## Abstract

Acne keloidalis nuchae (AKN) is a chronic scarring folliculitis that affects the occipital area. It is characterized by papule and pustule formation which eventually leads to tumor-like mass. Early cases can be managed by medical treatment that may require months, and, usually, lesions recur. In more severe cases, surgical excision is the mainstay of treatment. In our case, we proposed a staged approach when dealing with advanced stages of AKN. This includes deep excision of the lesion down to deep subcutaneous tissue with application of negative-pressure wound therapy (NPWT) for a week followed by resurfacing of the resultant defect with a split-thickness skin graft and NPWT for another week. This approach achieved quicker wound healing with no recurrence compared with other techniques such as healing by secondary intention. To our knowledge, this case is one of the most extensive cases published in the literature.

## Introduction

Acne keloidalis nuchae (AKN) is a chronic form of folliculitis and perifolliculitis. It affects the nape area and results in multiple itchy papules that easily get infected resulting in cicatricial alopecia and keloid-like plaque [1]. AKN is a misnomer as acne is not the cause and there is no true keloid formation. Moreover, the concomitant presence of keloid elsewhere is quite rare. AKN is more common among males of African descent. It was first described by Kaposi in 1869 as “dermatitis papillaris capillitii” [2]. Many theories have been postulated for its etiology, with some describing it as a mechanical form of folliculitis. Other theories relate it to metabolic syndrome [3].

Multiple modalities of treatment have been described for AKN including topical or intralesional steroid, retinoid, a prolonged course of oral antibiotics, especially rifampicin and clindamycin, and radiation. Surgical options include excision and direct closure, excision with healing by secondary intention, excision and immediate grafting, and use of tissue expander. Yet, achieving acceptable results and disease clearance is challenging [4].

Here, we present a two-stage approach in a severe case that showed good disease control with no evidence of recurrence after a long period of follow-up.

## Case presentation

**Figure 1:**
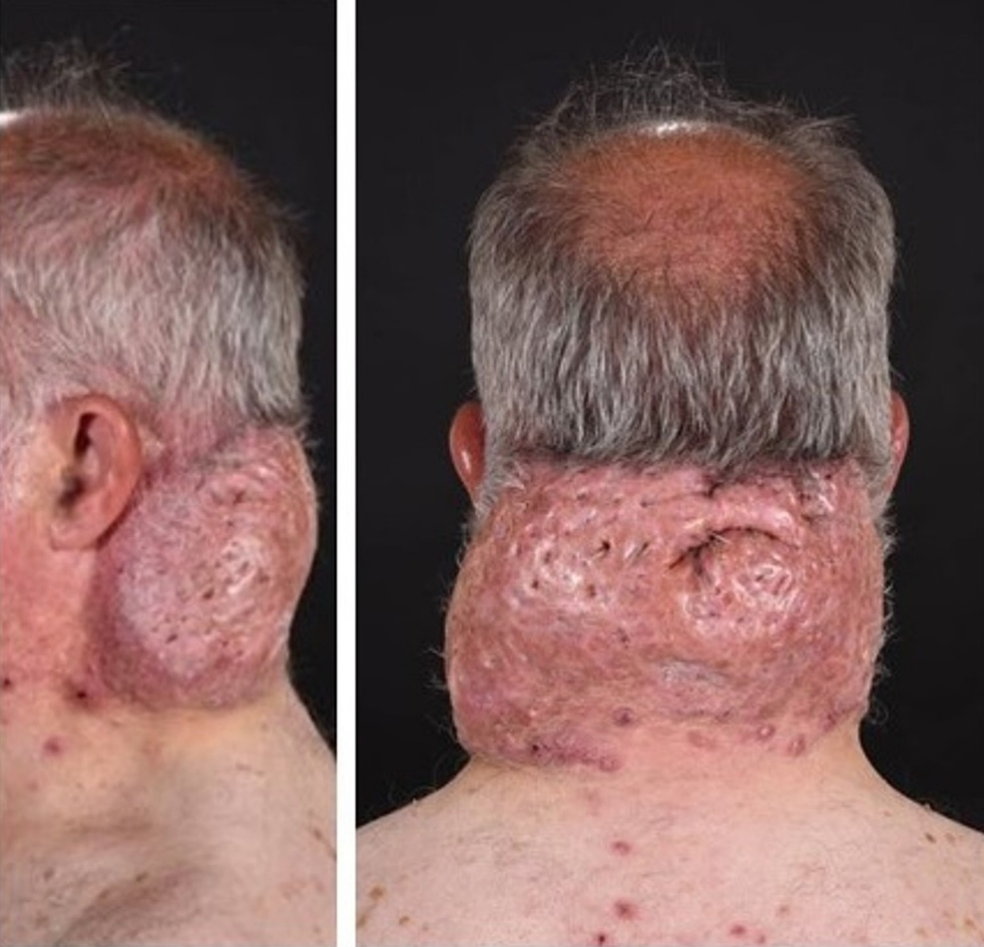
Preoperative photo showing the swelling of the entire occipital area.

A 58-year-old, white, non-smoker gentleman with a history of type 2 diabetes mellites presented to the Plastic Surgery Department of St. Georges’s Hospital with a 12-month history of small papules in the nape area which he started to notice after changing his barber. His general physician had tried a one-month course of oral antibiotics with no response. On assessment, almost the entire occipital scalp was replaced by a huge swelling with secondary infection (Figure 1).

**Figure 2:**
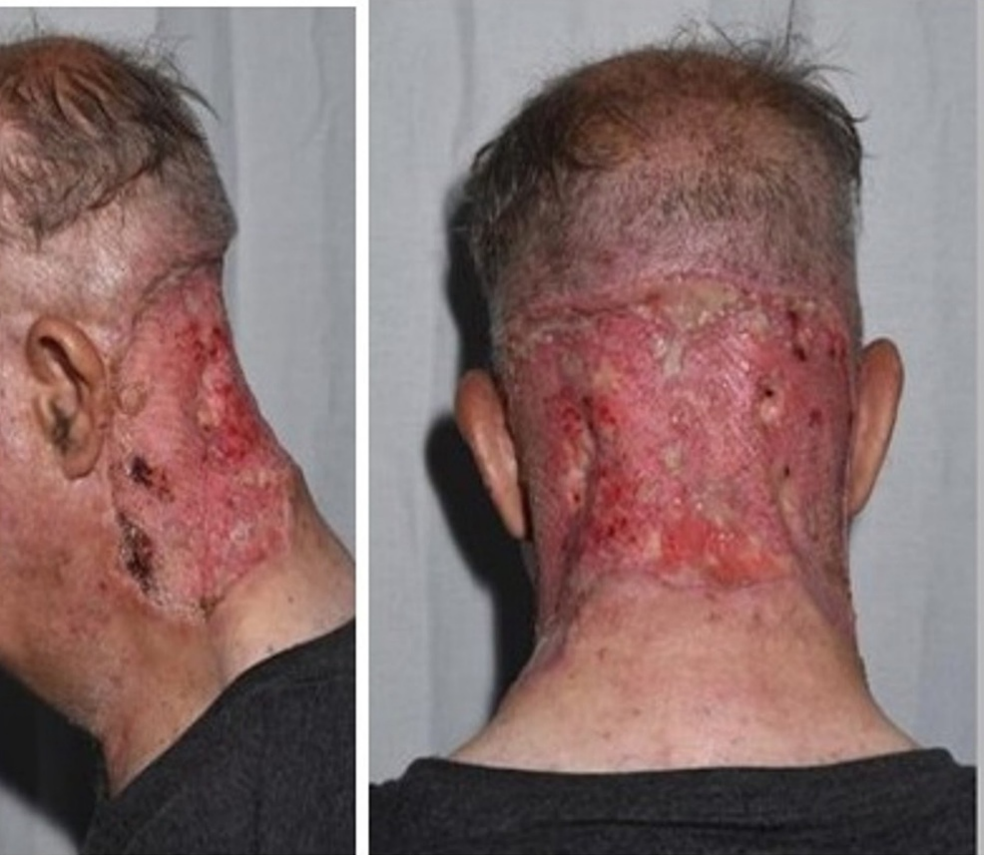
Second-week postoperative photo.

An oral course of rifampicin and clindamycin was prescribed resulting in good control of secondary infection. Moreover, dapsone and isotretinoin were tried based on the advice of a dermatologist for one year with little benefit. The patient opted for surgical intervention to resolve the disfiguring swelling in the occipital area. The operation was done in two stages. The first stage included excision of the entire lesion and application of negative-pressure wound therapy (NPWT). One week later, the patient underwent a split-thickness skin graft (SSG) and NPWT was reapplied. The postoperative period was uneventful except for the need for postoperative blood transfusion due to significant blood loss intraoperatively and over granulation tissue which was managed by topical steroid cream (Figure 2).

**Figure 3:**
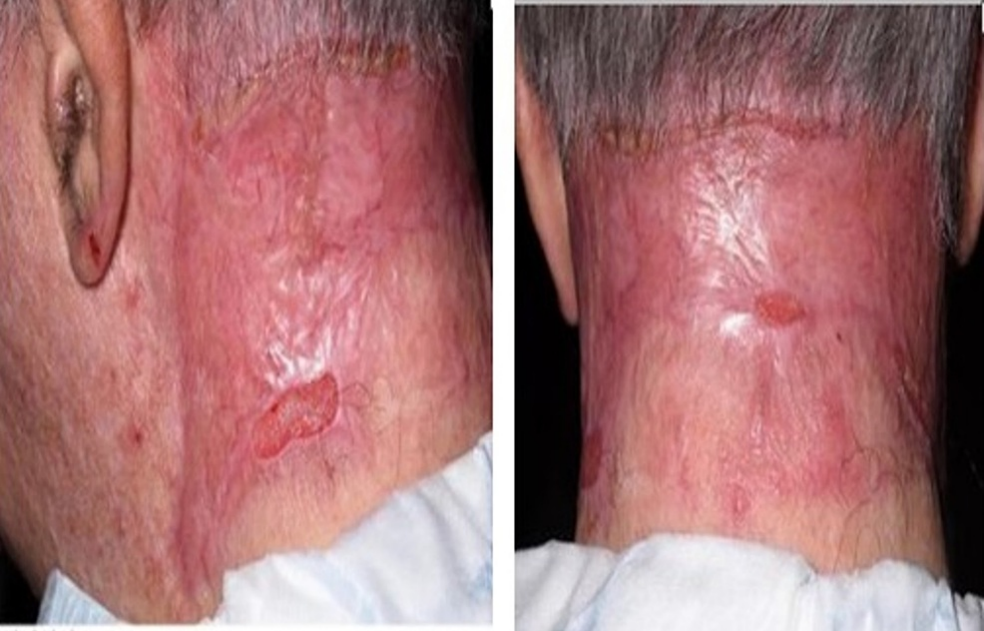
Eighth-month postoperative photo.

Eventually, all areas healed well within less than eight weeks of graft placement with no recurrence after follow-up for eight months (Figure 3).

## Discussion

AKN causes significant morbidity among affected individuals. Scarring, alopecia, and bad smell from recurrent bacterial infection are the most common complaints. Although the pathophysiology is unknown, possible etiologies include close shaving of hair in the nape area, constant mechanical irritation from shirt collars, and chronic low-grade bacterial infection. It may also present as a cutaneous manifestation of metabolic syndrome [3,5].

In early disease stages, lifestyle modification such as weight loss, avoidance of low haircuts, and avoidance of high-collared shirts along with medical treatment may be helpful. Medical treatment includes topical or intralesional steroids, topical or oral antibiotics, and/or oral isotretinoin. However, more advanced cases rarely respond to these measures. Radiotherapy has a limited role as it may increase the risk of skin cancers and head and neck malignancy, especially in large lesions that may necessitate larger doses of radiation [6].

Surgical excision is the mainstay in the management of advanced cases. Studies have suggested that subfollicular destruction decreases the risk of recurrence; hence, surgeons should aim deep excision down to the deep subcutaneous tissue or even the fascial layer [7]. Reconstructive options after excision include primary closure, secondary intention, or resurfacing by SSG. Primary closure is difficult to achieve in large areas as it may restrict head movement and lead to wounding dehiscence and widening of the scar later on. A secondary intention is another valid option after surgical excision, but prolonged healing of up to 12 weeks in severe cases remains an obstacle [4]. On the other hand, primary resurfacing by SSG carries a higher risk of graft failure as the wound bed may not be suitable for immediate grafting. Pestalardo et al. [8] reported the use of tissue expander in the initial stage, followed by excision of the lesion and advancement of the expanded flap to cover the resultant defect. They managed to control disease progression with no recurrence. Nevertheless, tissue expander complications such as infection and exposure are limiting factors and have been reported [8].

We propose a staged approach that includes primary deep excision down to the deep subcutaneous tissue along with the application of NPWT for one week, followed by resurfacing using SSG and NPWT for another week. We managed to achieve 80% wound healing by two weeks with small areas of over granulation which were managed by topical steroids. Complete wound healing was achieved in less than two months. Further, follow-up at eight months showed no evidence of any new lesions.

Following this staged approach saves the need for prolonged complicated dressing with the discomfort of oozing from a large wound, as in the case of secondary intention. It also improves the status of the wound bed which further increases graft uptake. One of the issues that we need to highlight in our case is that scalpel excision may lead to significant blood loss. Therefore, excision using electrosurgical cautery may be useful [9].

## Conclusions

AKN is a challenging condition to treat. Early stages can be managed by medical treatment while surgical excision is the treatment of choice for advanced disease. A staged excision is a useful tool in the armamentarium for the management of AKN, especially in extensive cases. It achieves quicker healing compared with secondary intention and decreases the chances of disease recurrence.
